# Wireless Peristaltic Pump for Transporting Viscous Fluids and Solid Cargos in Confined Spaces

**DOI:** 10.1002/adfm.202405865

**Published:** 2024-06-02

**Authors:** Saksham Sharma, Laura Caroline Jung, Nicholas Lee, Yusheng Wang, Ane Kirk-Jadric, Rishi Naik, Xiaoguang Dong

**Affiliations:** Department of Mechanical Engineering, Vanderbilt University, Nashville, TN 37212, USA; Department of Mechanical Engineering, Vanderbilt University, Nashville, TN 37212, USA; Department of Mechanical Engineering, Vanderbilt University, Nashville, TN 37212, USA; Department of Mechanical Engineering, Vanderbilt University, Nashville, TN 37212, USA; Vanderbilt Institute for Surgery and Engineering, Vanderbilt University, Nashville, TN 37212, USA; Department of Mechanical Engineering, Vanderbilt University, Nashville, TN 37212, USA; Vanderbilt School of Medicine, Vanderbilt University, Nashville, TN 37240, USA; Department of Mechanical Engineering, Vanderbilt University, Nashville, TN 37212, USA; Vanderbilt Institute for Surgery and Engineering, Vanderbilt University, Nashville, TN 37212, USA; Department of Biomedical Engineering, Vanderbilt University, Nashville, TN 37212, USA; Department of Electrical and Computer Engineering, Vanderbilt University, Nashville, TN 37212, USA

**Keywords:** esophageal stent, magnetic actuation, peristaltic pump, soft robot

## Abstract

The transport of fluids and solids is a vital process inside the human body, facilitated by the wave-like motion in the lumen called peristalsis. However, peristalsis may be compromised due to tumor growth, resulting in difficulties in lumen motility. The dysmotility of the human lumen can result in blockages and pose numerous challenges, including aspiration in the lungs and reproductive issues in the female oviduct. Restoring peristalsis in medical devices, such as medical stents, can prevent device blockage and promote effective transport. Here, a wirelessly actuated soft robotic undulating pump designed to efficiently transport both viscous fluidic and solid cargos is proposed. The kinematics of the single sheet and the coordination between pairs are systematically designed to generate undulation and peristalsis, enabling the pumping of both liquids and solids. The integration of the undulating pump is demonstrated onto an esophageal stent. The same undulating motion-based pumping mechanism can be adapted for usage in other organs, such as the female oviduct, thereby offering potential applications for treating lumen dysmotility in various diseases. The proposed wirelessly actuated robotic pumping mechanism holds promise in facilitating diverse implantable medical devices aimed at treating diseases characterized by impaired peristalsis and dysmotility.

## 1. Introduction

Within the human body, the transport of viscous fluids and solids plays a pivotal role in various biological process facilitated by the peristalsis^[1]^ within luminal structures like the esophagus,^[2]^ small intestine,^[3]^ and female oviduct.^[4]^ The peristalsis is a wave-like movement of the muscles in the human lumen to efficiently move viscous fluids and solid cargos.^[3,5-6]^ The impairment of peristalsis in various diseases can result in luminal occlusion. Particularly, tumors in the esophagus may grow and obstruct the lumen, hindering the passage of food and liquids. Particularly, esophageal adenocarcinoma is rising in prevalence and primarily affects the aging population, with the median age of patients diagnosed between 65 and 74 years old, and over 30% of cases occurring in individuals over the age of 75 in the United States.^[7]^ In other diseases, such as achalasia, the dysmotility of the esophagus leads to food blockage when the esophageal muscles fail to contract properly, preventing the downward movement of food toward the stomach.

Treatment often involves the usage of a silicone or metal tube known as an esophageal stent^[8,10]^ to maintain an open passage in the narrowed portion of the esophagus, to allow the swallowing of solids and liquids. However, challenges arise when food particles or other materials accumulate around the stent, leading to blockages, and in some cases aspiration when the food enters the airway.^[11]^ This obstruction may cause the ingested material to divert into the airways instead of progressing into the stomach, which poses a significant concern, particularly for individuals with compromised respiratory function. It can result in respiratory complications such as pneumonia. Therefore, restoring the peristalsis is crucial for the effective transport of liquids and solids, addressing these challenging medical conditions.

The development of a peristalsis-based soft robotic pump holds promise for mimicking and restoring peristalsis.^[12]^ Compact and wirelessly actuated soft robotic pumps designed for transporting cargos inside medical stents could potentially overcome these challenges. Soft robotic pumps, powered by various soft actuators such as fluidic and pneumatic actuators,^[13]^ electropneumatic,^[14]^ dielectric elastomer actuator,^[15-17]^ and electrodynamic effect^[16,18]^ been shown before but have not been demonstrated to integrate into medical stents due to several fundamental challenges. First, soft robotic pumps, propelled by fluidic^[19]^ or pneumatic^[13,20]^ means, face the hurdle of requiring bulky and tethered external compressors, posing integration challenges for medical stents. Second, although electrically driven pumps show promise with their compact size,^[21]^ the batteries required for onboard power and actuation currently have severely limited lifetimes.^[22,23]^ Additionally, the incorporation of flexible electronics to regulate actuator motion while maintaining resilience and conformity to curved surfaces presents significant challenges in circuit design and fabrication.

In contrast, external actuation methods such as acoustic,^[24,25]^ magnetic actuation,^[26-29]^ and chemical actuation,^[30]^ offer a non-invasive method to actuate soft structures to pump fluids by penetrating biological tissues deep inside the body. Among them, magnetic actuation could penetrate deep inside the organs and provides larger mechanical forces and torques when combined with ferromagnetic or ferrimagnetic materials.^[26,31,32]^ Existing works have shown arrays of millimetre- or micrometer-scale magnetic soft actuators for transporting microparticles in liquids.^[33-37]^ However, the current pumping mechanism is limited to lab-on-a-chip fluid transport and have not been shown to be integrated into medical stents. In addition, the design and control of magnetic structures that allow efficient pumping mechanism for viscous fluids and solids remains unclear.

To tackle this challenge, we propose a magnetically actuated soft robotic pump that can restore peristalsis through the incorporation of magnetically actuated soft sheets within the stent structure. Wireless actuation using external magnetic fields eliminates the need for bulky on-board components, ensuring the safe interaction of the soft robotic structure with esophageal tissue. We systematically investigate both single sheet pumping and pair-wise coordination of the magnetic soft sheets to optimize the pumping efficiency. The soft robotic pump could transport viscous fluids, particle-liquid suspensions, biofluids such as mucus, and solid spheres of different sizes. Finally, we demonstrate integrating the undulating pump into a silicone and metal stent for the esophagus to generate artificial peristalsis to aid in propelling liquids and solids inside an esophageal phantom. The active pumping function is shown to reduce the risks of the blockage of the lumen in the control experiments. The same pumping mechanism can be potentially used for patients with diseases of dysmotility in various lumens inside the body. Our proposed mechanism of restoring the natural peristalsis motion thus paves the way for the next-generation robotic medical devices to improve the quality of life.

## 2. Results

### 2.1. Concept, Design, and Fabrication of the Undulating Motion-Based Pump

**Figure 1.**
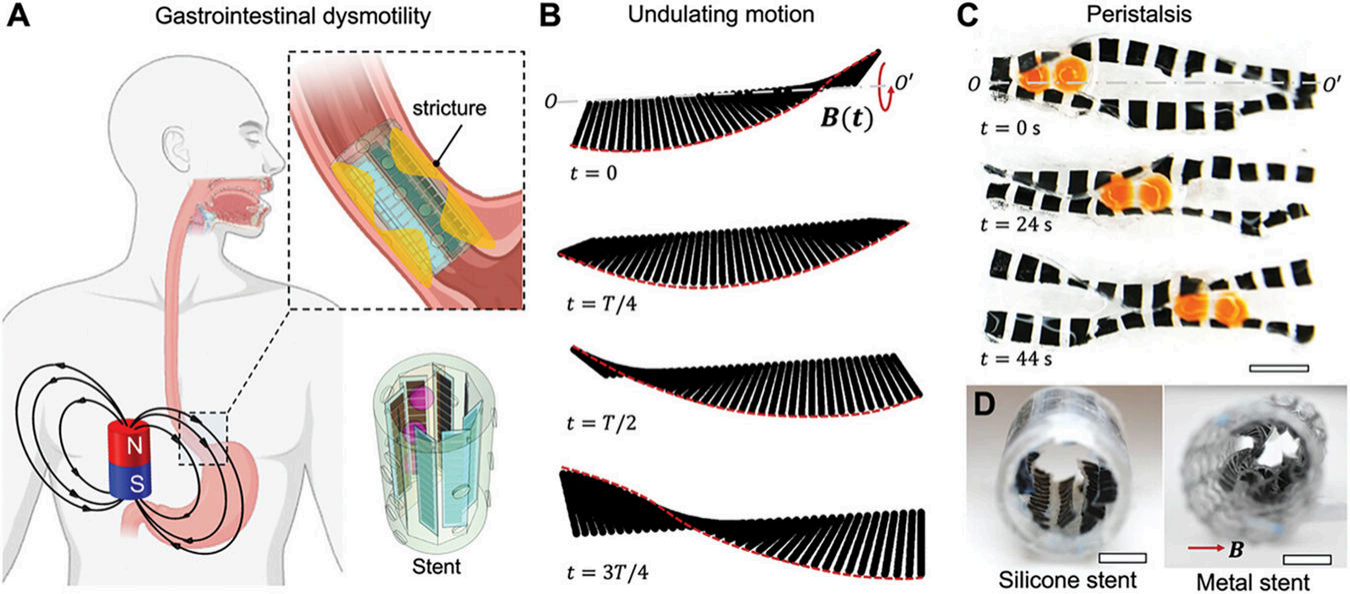
Concept of the wirelessly actuated undulating pump and its integration into an esophageal stent. A) Concept of an esophageal stent with integrated magnetic soft sheets in the upper gastrointestinal tract for restoring peristalsis. A rotating external magnetic field is applied to actuate the magnetic soft sheets. B) Illustration of the undulating motion of a magnetic sheet within a period driven by a rotating external magnetic field. The magnetic field is rotating about the fixed long edge of the magnetic soft sheet. One long edge of the magnetic sheet is fixed while the other edge is free. C) Sequential optical images of a pair of magnetic soft sheets transporting solid spheres. Scale bar, 5 mm. D) Illustration of an esophageal stent integrated with the magnetic soft sheets transporting both liquids and solids. Scale bars, 5 mm.

**Figure 2.**
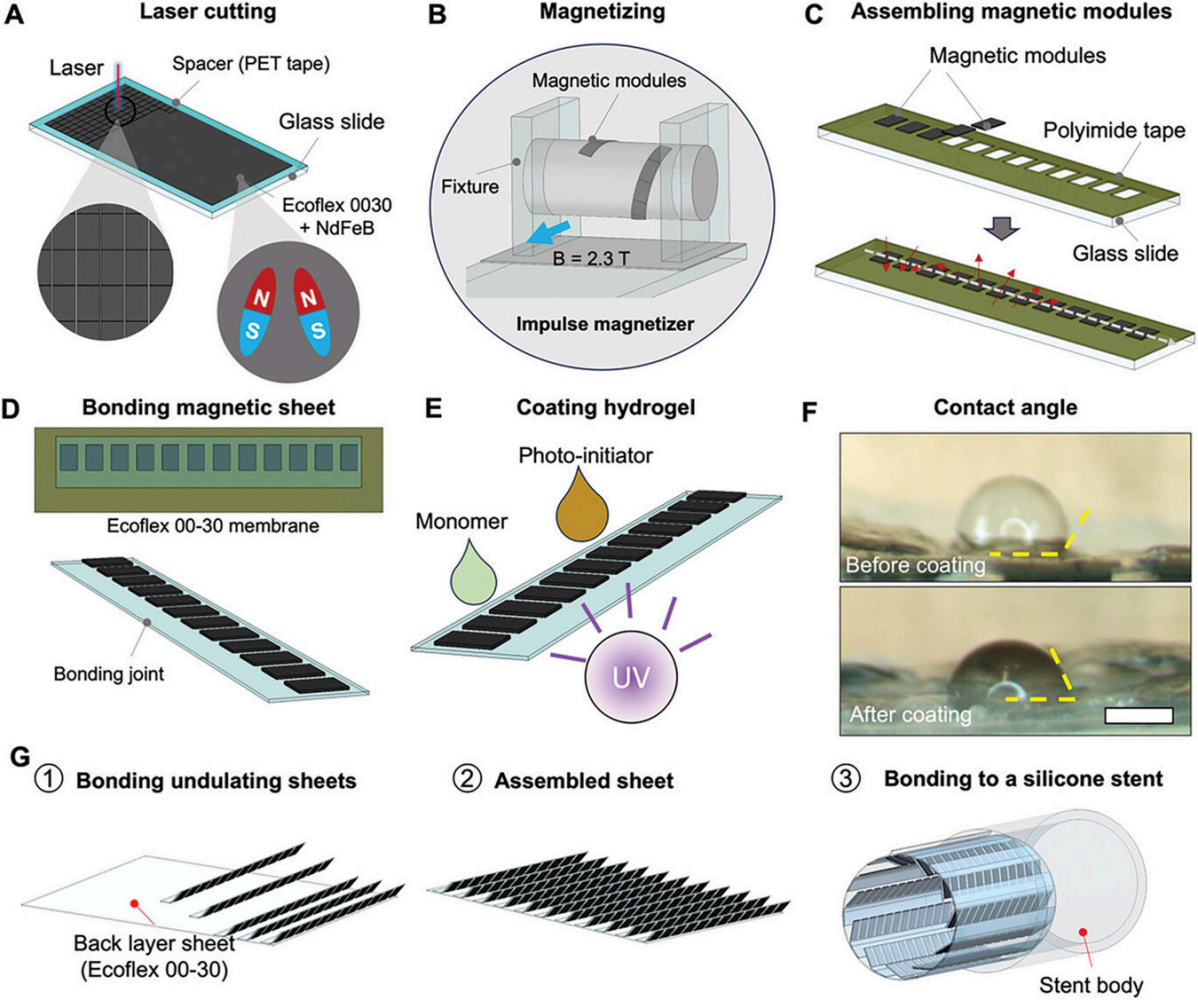
Fabrication and characterization of the magnetic soft sheet. A) Illustration of fabricating the magnetic modules by cutting a thin sheet of magnetic composite using a UV laser machine. The thin sheet is made of NdFeB particles and Ecoflex 00–30 (weight ratio: 2 to 1) with thickness controlled by a Polyester tape as a spacer. B) Illustration of magnetizing the magnetic modules inside an impulse magnetizer (IM-10-30, ASC Scientific). The magnetic modules are wrapped around a cylinder to encode the desired magnetization profiles. C) Illustration of assembling the magnetic modules into a magnetic soft sheet with the assistance of a fixture made of a Polyimide tape. Each magnetic module is 3 mm by 1.5 mm by 0.2 mm. D) Illustration of bonding the magnetized modules to an elastic membrane made of Ecoflex 00–30 with a thickness of 200 μm. E) Illustration of coating a thin layer of Polyethylene Glycol Diacrylate (PEGDA) hydrogel on the magnetic sheet to reduce adhesion. F) Images of the uncoated and coated sheets with measured water contact angles (104 and 65 degrees). Scale bar, 500 μm. G) Illustration of assembling multiple magnetic sheets into a cylindrical pump (silicone stent).

The undulating motion of a magnetic soft sheet plays a fundamental role in pumping liquid and solid cargos in the proposed wireless soft robotic pump. As shown in Figure 1A, the objective is to produce peristalsis through programming an array of magnetic soft sheets and controlling their coordinated movements using external magnetic fields. As illustrated in Figure 1B, the application of a rotating magnetic field B(t) induces the undulating motion in each sheet. A magnetic soft sheet consists of magnetic modules created by blending ferromagnet particles (NdFeB, average diameter: 5 μm) in a polymer matrix, and magnetized in a specific magnetization profile (see Figure 2 and “Experimental Section” for the fabrication process). The distributed magnetic torque induced along the sheet long axis *OO*′ creates the undulating motion by bending the magnetic modules along the axis *OO*′ of the sheet. When subjected to an external magnetic field, a pair of magnetic sheets will have coordinated motion like peristalsis for transporting particles.

The pairs of magnetic soft sheets are further integrated into a medical stent to allow transporting liquid and solid cargos as illustrated in Figure 1C. By integrating the magnetic soft sheets in a circular array within a stent, i.e., a hollow tube, it serves a dual purpose: providing radial support to the lumen in cases of stricture while simultaneously restoring the peristalsis motion for pumping. Particularly, an esophageal stent is designed to assist in the digestion of large food pieces, thereby preventing blockages and aspirations. The coordination between neighboring sheets and the surface of the hollow tube creates a peristalsis motion, showcased for transporting liquids and solids. The wireless actuation capability of the magnetic soft sheets allows for minimally invasive cargo transport. Notably, magnetically actuated devices have been reported before with wireless actuation and small sizes in lab-on-a-chip applications^[33,34,27]^ and miniature soft robots that can swim or crawl^[38,39]^ for minimally invasive medical operations. However, magnetically actuated soft robotic pumps that can restore peristalsis and seamlessly integrated with medical stents have not been reported before.

In Figure 2, we further introduce the method of fabricating the magnetic soft sheets. The fabrication process includes preparing the magnetic modules, programming their magnetization, and assembling them into magnetic soft sheets, and integrating into a medical stent (see Materials and Methods for the details). Briefly, first, the magnetic modules (dimensions: 3 mm by 2 mm by 0.2 mm) are prepared by cutting a rectangular magnetic sheet made of NdFeB micromagnets and Ecoflex 00–30 by a laser machine as shown in Figure 2A. Second, the magnetic modules in a magnetic soft sheet are assembled into a strip in a head-to-tail manner and magnetized by wrapping the strip around a cylindrical fixture in an impulse magnetizer with a magnetic field of 2.3 T as illustrated in Figure 2B. Third, the magnetized magnetic modules are assembled in a side-by-side manner with the assistance of an assembly jig made from a polyimide tape (Figure 2C). Fourth, the magnetic modules are further bonded to a thin layer of Ecoflex 00–30 and released from the assembly jig (Figure 2D). Lastly, the assembled structure could be further coated with other materials such as PDMS and hydrogel for biocompatibility and hydrophilic surface property to facilitate liquid wetting. Figure 2E demonstrates one example of coating Polyethylene Glycol Diacrylate (PEGDA) hydrogel on the surface of the device. The water contact angle is significantly reduced from 104 degrees to 65 degrees after the hydrogel coating that allows better wetting of water-based liquid as a lubrication layer as shown in Figure 2F. Multiple magnetic sheets that are obtained with the same method are further bonded to a thin back layer made of Ecoflex 00–30 and then wrapped into a tube to be further bonded (Figure 2G) to the inside surface of an elastic cylindrical tube, a silicone stent, and other meshed metal stents.

### 2.2. Single Magnetic Sheet Undulating Motion

**Figure 3.**
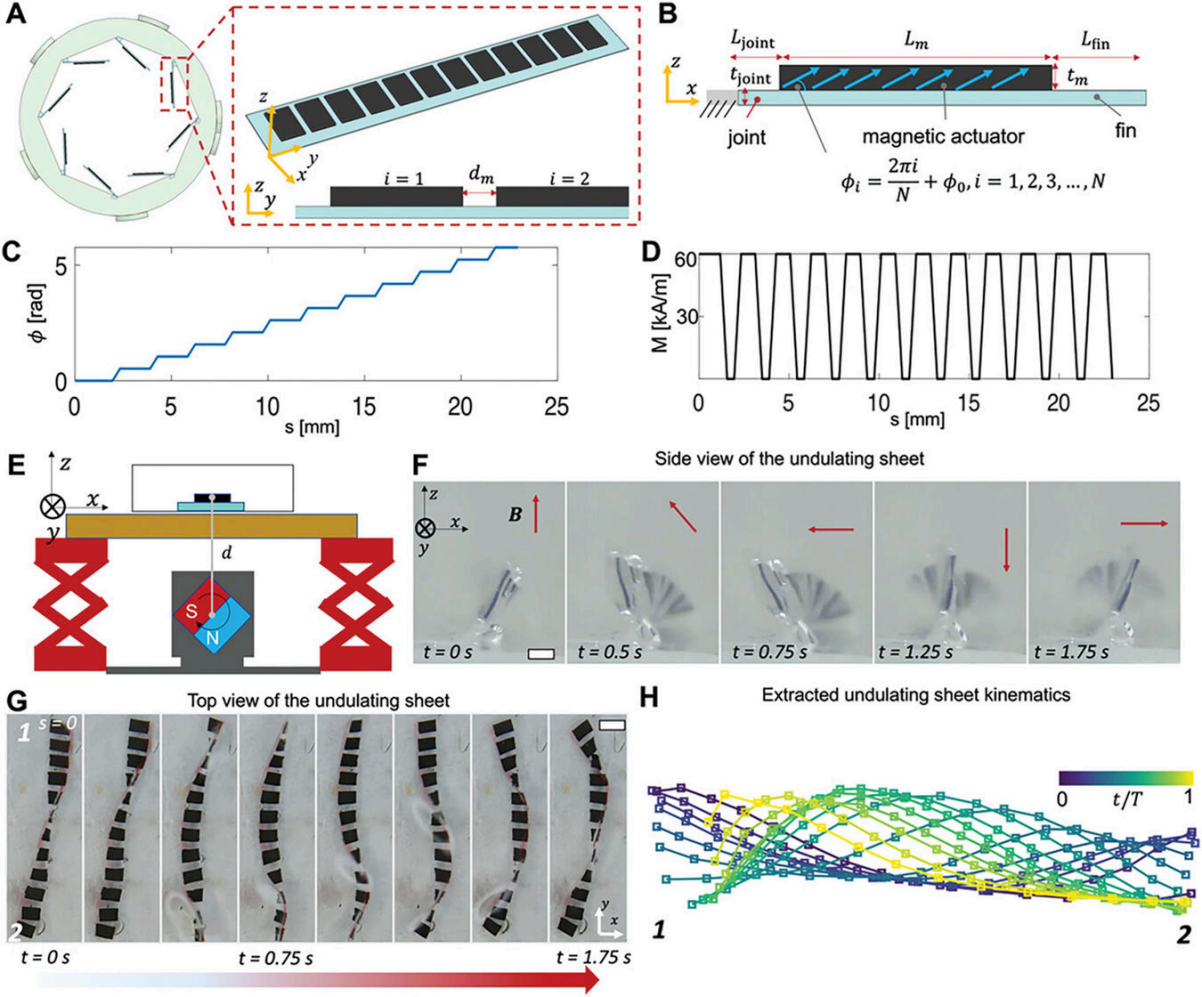
Design and characterization of a single magnetic soft sheet. A) Illustration of the magnetization profile of a magnetic soft sheet in a circular array. For each sheet, the magnetization phase profile is defined as $\phi_{i}(s)$, s∈(0,L]. B) Illustration of the magnetization and dimension of a magnetic module. C,D) The magnetization phase (C) and magnitude (D) profiles for a magnetic soft sheet. E) Illustration of the magnetic actuation setup for the experiments in (F,G). F) Video frames (Movie S1, Supporting Information) of a magnetic soft sheet under a rotating magnetic field in the x-z plane. External magnetic field: average magnitude *B* = 55 mT, frequency *f* = 0.5 Hz. G. Video frames (Movie S1, Supporting Information) of a magnetic sheet under a rotating magnetic field in the x-y plane. External magnetic field: average magnitude *B* = 55 mT, *f* = 0.5 Hz. H. The extracted kinematics of the magnetic soft sheet shown in (G). In (F,G), the liquid is glycerol (dynamic viscosity: 983 mPa s).

The undulating motion of a single magnetic sheet is achieved by patterning its magnetization profile. As shown in Figure 3A, the magnetic soft sheet is designed to have multiple magnetic modules with a rotational angular offset encoded between neighboring magnetic moments in the x−z plane. Figure 3B shows that the angle of the magnetic moment $M_{i}$ for the i-th (i=1,…,N) magnetic module is given by $\phi_{i}=\frac{2\pi i}{N}+\phi_{0}$, where $\phi_{0}$ is the initial phase of the magnetic module. $M_{i}$ is then given by $M_{ix}=M\cos\phi_{i}$, $M_{iz}=M\sin\phi_{i}$ (magnetization magnitude: *M* = 61.9 kA m^−1^). The magnetization phase and magnitude profiles given in Figure 3C,D are essential to produce the undulating motion. When a rotating magnetic field B(t) is applied in the x−z plane, each magnetic module experiences a magnetic torque that bends the module at an angle. The magnetic torque applied on each magnetic module is given by $\tau_{i}=V_{m}M_{i}\times B(t)$ where $V_{m}$ is the volume of the magnetic module. There is also fluid drag applied on the sheet that is depending on the rotating frequency of the magnetic field *f* and the dynamic viscosity of the liquid μ. The bending motions of the neighboring magnetic modules are coupled due to the connection with non-magnetic materials. The bending angle is shifted across different magnetic modules along the sheet’s long axis. Jointly they create an undulating motion that travels along the sheet as the magnetic field rotates in clockwise or counterclockwise in the x−z plane. Compared with other types of undulating pumps,^[40]^ the reported magnetic sheets are fully soft, wirelessly actuated, and have a relatively good programmability in terms of the pair-wise coordination by jointly designing neighboring sheets.

To further demonstrate the undulating motion, Figure 3E-H showcase a magnetic sheet submerged inside glycerol when being actuated by a rotating magnetic field with an average magnitude of *B* = 55 mT, and frequency of *f* = 2 Hz (Figure S1, Supporting Information). A rotating permanent magnet (50 mm by 25 mm by 25 mm, NdFeB, N45) is used to actuate the magnetic sheet as shown in Figure 3E (see Figure S2, Supporting Information, for the experimental setup). Subsequently, Figure 3F,G show the optical images of the magnetic soft sheet in a side view and top view, respectively. Traveling wave motions are clearly shown in both the top view and the side view. Further extraction of the curve of the sheet edge shows the quantitative characterization of the traveling wave like motion as shown in Figure 3H, when the peak value of the curve is shifting along the *y*-axis in a period. The extracted curves show that a peak shift from one end “1” to the other end “2”, indicating a traveling wave propagating as the magnetic module bends sequentially with a phase lag. The phase lag is fully programmable by designing the magnetization of each magnetic module. Lastly, with the flexible connection between neighboring magnetic modules, the traveling wave is induced that is essential for the efficient propulsion of liquids and solids.

### 2.3. Pair-Wise Sheet Coordination for Pumping Liquids and Solids

**Figure 4.**
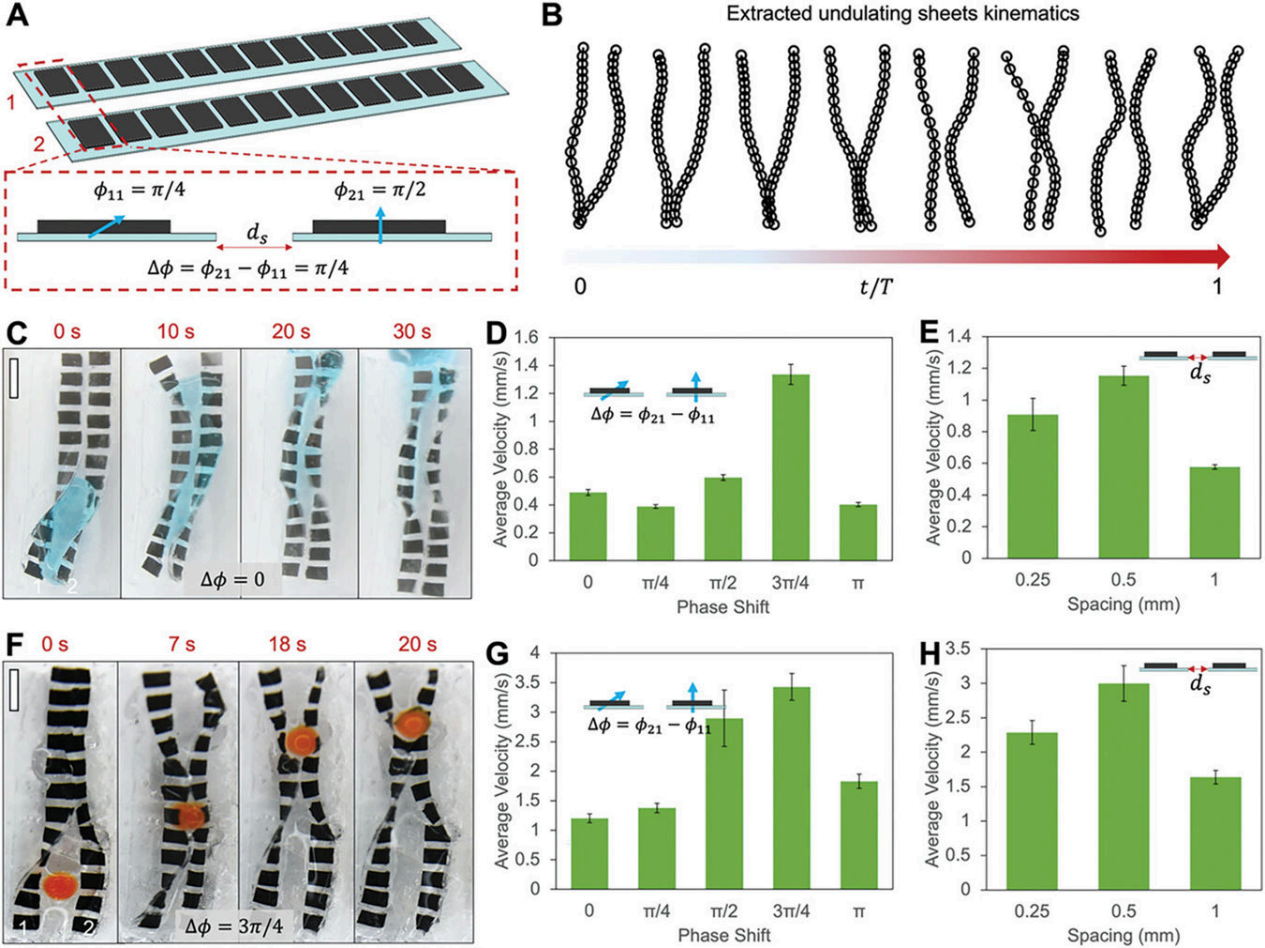
Characterization of a pair of magnetic soft sheets for pumping. A) Illustration of a pair of magnetic soft sheets. The phase shift between two magnetic soft sheets is defined as the angle difference of the magnetic moments of the front magnetic modules $\Delta \phi =\phi_{21}-\phi_{11}$. The sheet spacing is denoted as $d_{s}$. B) Extracted kinematics of a pair of magnetic soft sheets with a phase shift of Δϕ=3π∕4. C) Video frames (Movie S2, Supporting Information) of transporting liquid (syrup) by a pair of magnetic soft sheets. Phase shift Δϕ=0. $d_{s}$ = 0.5 mm. Average *B* = 55 mT, *f* = 2 Hz. D) The average liquid transporting speed as a function of the phase shift Δϕ. Magnetic field: *B* = 55 mT, *f* = 2 Hz. E) The liquid transporting speed as function of sheet spacing. In (C–E), the liquid is syrup with a dynamic viscosity of 5000 mPa s. F) Video frames (Movie S2, Supporting Information) of transporting a solid sphere (hydrogel bead) by a pair of magnetic soft sheets. Phase shift Δϕ=3π∕4, $d_{s}$ = 0.5 mm. Magnetic field: *B* = 55 mT, *f* = 2 Hz. G) The average bead transporting speed as a function of the phase shift between two neighboring sheets. H) The average bead transporting speed as a function of sheet spacing. In (F–H), the diameter of the hydrogel bead is 3.5 mm. In (F–H), the liquid for lubrication is glycerol. Error bar indicates standard deviation for n = 5 trials. In all figures, scale bars, 5 mm.

We further show the kinematics of a pair of magnetic sheets by varying their phase difference in Figure 4 and Movie S2 (Supporting Information). Two magnetic sheets could be designed differently to induce coordinated motion for generating peristalsis. The coordinated motion is due to the time lag between the two neighbouring sheets with different magnetization profiles undergoing undulating motion. Although the two magnetic sheets experience similar temporal magnetic torques and undulating motion, the phase in their motion depends on both the planted orientation and their magnetization profile when actuated by the same magnetic field. The two magnetic sheets have different magnetization phase $\phi_{11}$ and $\phi_{21}$ as shown in Figure 4A. With a constant phase difference $\Delta \phi =\phi_{21}-\phi_{11}$ (e.g., Δϕ=3π∕4) for all magnetic modules, pair-wise coordination is induced as shown in Figure 4B. The extracted kinematics in Figure 4B shows that a narrow part is traveling when rotating the external magnetic field, which creates peristalsis.

To further investigate the pumping performance of a pair of magnetic sheets of different designs, we study the liquid and solid pumping speeds when varying the phase difference and spacing of a pair of magnetic sheets. Figure 4C shows that a liquid droplet (syrup, dynamic viscosity: 5000 mPa s) with a volume of ≈1 mL is effectively transported by a pair of magnetic sheets from one side to the other within 30 s. In Figure 4D, we further compare the liquid droplet pumping speed by several different pairs of magnetic sheets which have a phase difference Δϕ from 0 to 3π∕4. The pair of sheets with Δϕ=3π∕4 allow the maximum average transporting speed of ≈1.3 mm s^−1^. The pair-wise motion in this case resembles the peristalsis when a narrow part between the two sheets is traveling along the long axis of the two sheets. Moreover, Figure 4E shows the average transporting speeds by magnetic soft sheet pairs with different spacings $d_{s}$, which is defined as shown in Figure 4A. For the sheets with a width of ≈3 mm, the maximum transporting speed is achieved when they have $d_{s}$ = 0.5 mm and Δϕ=3π∕4. The magnetic interaction becomes negligible at a separation distance of $d_{s}$ = 0.5 mm. This is because the magnetic field generated by a neighboring magnetic module typically registers <1 mT, notably lower than the magnetic field produced by external magnets, which typically measures ≈55 mT. When further decreasing the spacing to 0.25 mm, the transporting speed is deteriorated by the magnetic interaction between the two magnetic sheets. In contrast, when further increasing the spacing to 1 mm, the transporting speed is also reduced when the coordinated peristalsis motion between the two sheets becomes less efficient.

Meanwhile, to investigate the performance of pumping solids using a pair of magnetic soft sheets, we prepare magnetic sheets of different phase shifts and spacings to perform solid pumping experiments. Figure 4F shows that a hydrogel solid sphere of a diameter of 3.5 mm is being transported from one end to the other when slightly lubricated by glycerol. The pair of sheets have Δϕ=3π∕4 and a spacing of $d_{s}$ = 1 mm. In addition, Figure 4G shows that a pair of sheets with Δϕ=3π∕4 gives the maximum transporting speed consistent with that in the liquid droplet transporting experiments. Moreover, Figure 4H shows that when the spacing is $d_{s}$ = 0.5 mm, the pair of magnetic soft sheets give the maximum transporting speed as the two sheets could exert a relatively large pressure on the solid sphere to propel it forward. Lastly, the hydrogel layer coated on the magnetic sheets provides a lubrication layer that greatly enhances the pumping of solid cargos as shown in Figure S3 (Supporting Information).

### 2.4. Demonstration of Integrating Magnetic Soft Sheets Inside a Silicone Tube for Pumping

**Figure 5.**
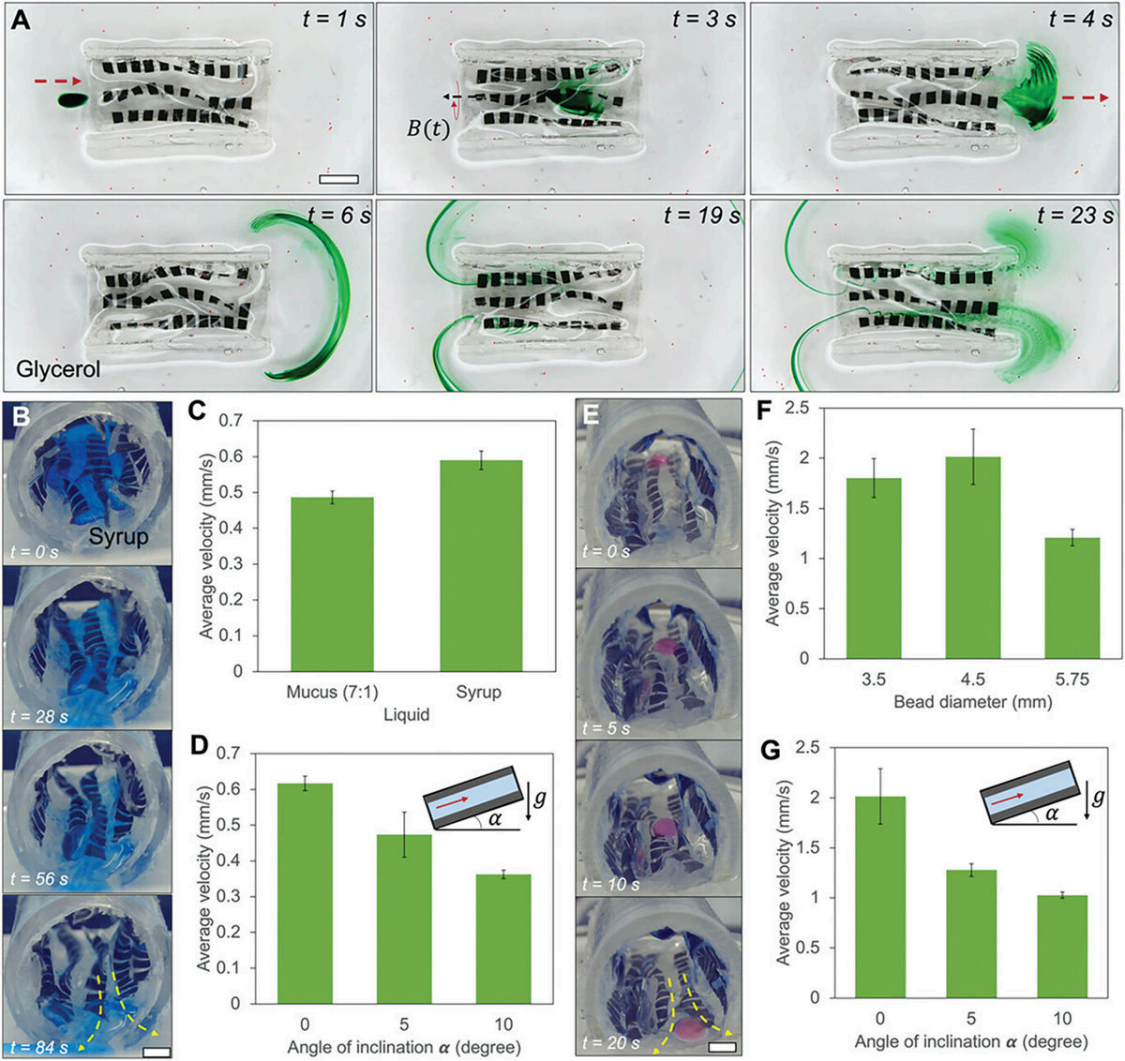
Characterization of the performance of transporting liquid and solid cargos by a cylindrical pump. A) Video frames (Movie S3, Supporting Information) of three magnetic sheets (phase shift: Δϕ=3π∕4) pumping in glycerol visualized by green dye. B) Video frames (Movie S3, Supporting Information) of pumping liquid (syrup) inside a silicone tube. C) The average transporting speed of mucus and pure syrup using the magnetic soft sheets inside a tube. D) The average transporting speed of syrup when the cylindrical pump is placed at different angles. E) Video frames (Movie S3, Supporting Information) of pumping hydrogel spheres by the cylindrical pump. F) The average transporting speed of hydrogel spheres in different diameters by the cylindrical pump. Glycerol is used as a lubricant liquid. G) The average transporting speed of hydrogel spheres (diameter: 4.5 mm) when the cylindrical pump is placed at different angles. In all experiments, magnetic field: *B* = 55 mT, *f* = 2 Hz. In all experiments, the dynamic viscosities of syrup and mucus are 5000 mPa s and 11 307 mPa s, respectively. Error bars indicate standard deviation for n = 5 trials. Scale bars, 5 mm.

The magnetic soft sheets with optimized phase difference and spacing are further integrated into a silicone tube as demonstrated in Figure 5 and Movie S3 (Supporting Information). With the optimized magnetic sheets in terms of both phase difference and spacing, we integrate ten magnetic sheets inside a tubular structure made of silicone to create a cylindrical pump for transporting liquids and solids. First, the fluid flow pattern is shown in Figure 5A which demonstrates the efficient pumping of viscous liquid (glycerol, dynamic viscosity: 890 mPa s) visualized by green dyes. The wake pattern when the stent is fully submerged in glycerol is shown in Figure S4 (Supporting Information). Second, Figure 5B shows an investigation of the pumping speed for different types of liquids. We test the pumping speed for syrup (dynamic viscosity: 5000 mPa s) and porcine mucus by mixing porcine mucin with water according to a mixing ratio of 7:1 by weight. The porcine mucus has a viscosity of ≈11 300 mPa s at room temperature. In both cases, the cylindrical pump shows a relatively fast pumping speed with ≈0.5 and 0.6 mm s^−1^ for the syrup and mucus, respectively, as shown in Figure 5C. The presence of biofluids such as mucus may allow better transportation as mucus is slippery as shown in Figure S5 (Supporting Information). Lastly, we tilt the cylindrical pump to see if it can overcome gravity to pump the liquids upward effectively. Figure 5D shows a reduced transporting speed for syrup when increasing the tilt angle from 0 to 10 degrees. The angle may be further increased when producing smaller and denser magnetic sheets inside the pump. Nonetheless, the demonstrated cylindrical pump inside a silicone stent shows promising applications of transporting viscous liquids.

Similarly, in Figure 5E-G, we investigate the ability to pump solids of different sizes and at different tilting angles using the cylindrical pump. Figure 5E shows that multiple hydrogel beads of different sizes are transported simultaneously. Moreover, Figure 5F shows that the beads with a diameter of ≈4.5 mm are transported at the fastest speed due to the relatively large pressure applied on the beads by the magnetic sheets, while the peristalsis motion is not deteriorated due to the sheet-solid interaction. Lastly, Figure 5G shows that the cylindrical pump can also pump solid spheres up to 10 degrees upward indicating its ability to overcome gravity for pumping solids.

### 2.5. Demonstration of Integrating Magnetic Soft Sheets Inside a Medical Stent for Pumping

**Figure 6.**
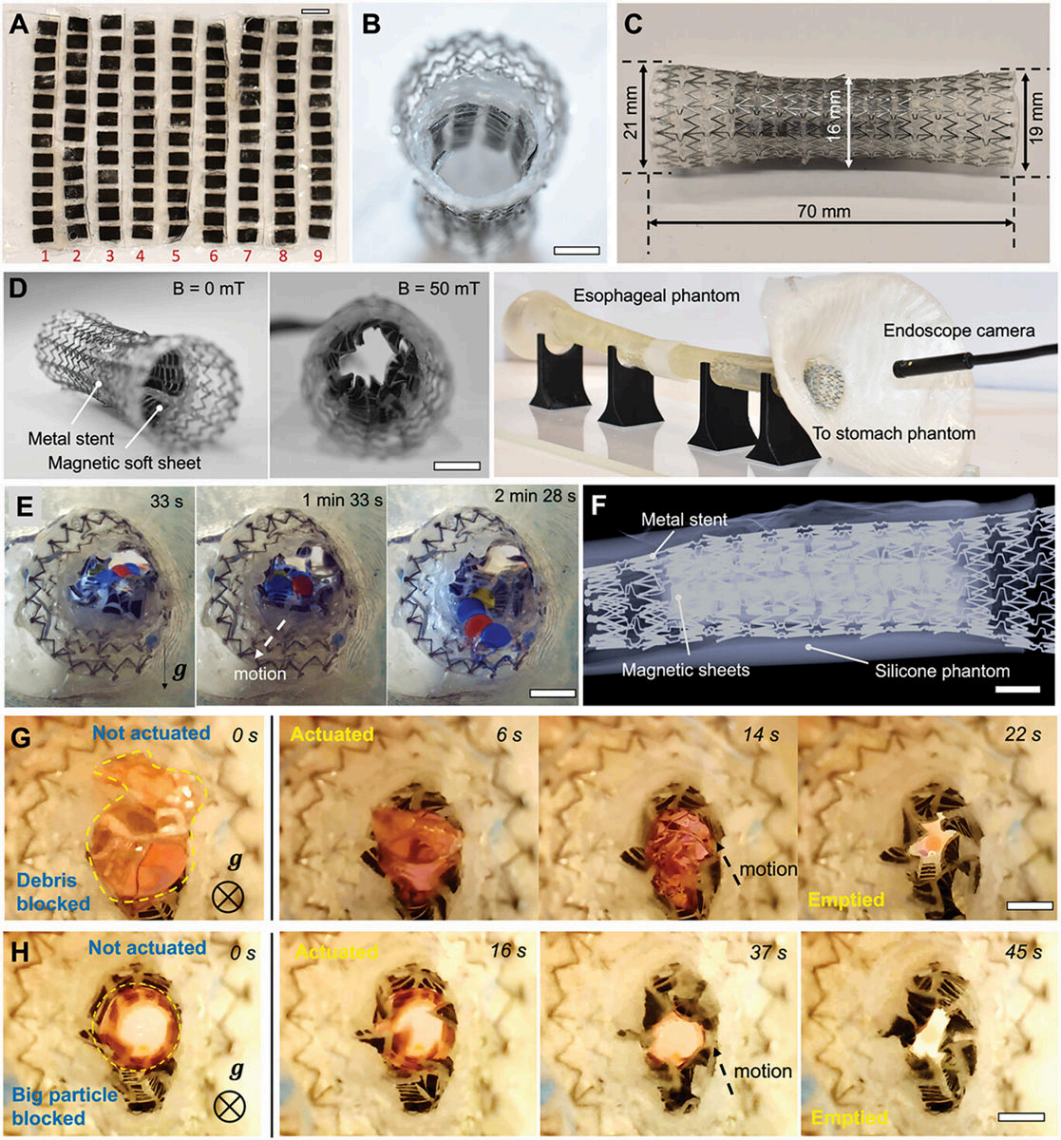
Demonstration of integrating magnetic soft sheets inside a metal stent for pumping liquids and solids. A) Optical image of the nine magnetic soft sheets. Each sheet has 12 magnetic modules. B) Optical image of the assembled metal esophageal stent with integrated magnetic soft sheets. C) Optical image of the assembled esophageal stent with marked dimensions. D) Experimental images of the metal esophageal stent with integrated magnetic soft sheets and the experimental setup used for testing. An external magnetic field of an average magnitude of ≈55 mT is applied. The system includes a magnetic actuation system, the esophagus phantom, the metal stent with integrated magnetic soft sheets, and an endoscope camera. E) Video frames (Movie S4, Supporting Information) of the metal stent transporting liquid (syrup, dynamic viscosity: 5000 mPa s) and hydrogel spheres inside an esophagus phantom. F) X-ray image of a metal esophageal stent inside the phantom. G) Video frames (Movie S4, Supporting Information) of transporting multiple hydrogel pieces by the stent. H) Video frames (Movie S4, Supporting Information) of transporting a large hydrogel sphere by the stent. Scale bars, 5 mm.

To further show the potential for medical applications, the undulating pump is demonstrated to integrate into a commercial metal stent (16 × 70 ALIMAXX-ES Fully Covered Esophageal Stent, Merit Medical) as shown in Figure 6 and Movie S4 (Supporting Information). We integrate nine magnetic sheets with the optimized phase difference and spacing inside an esophageal stent as shown in Figure 6A-C. The stent is already covered with a polymer mesh as shown in Figure 6B, which is for preventing stent tissue ingrowth. Built on that, our magnetic sheets are first wrapped into a cylindrical pump and then the cylindrical pump is further bonded to a metal stent (see Materials and Methods for details). We deploy the assembled meshed metal stent by squeezing it to fit inside a 3D-printed esophagus phantom as shown in Figure 6D. The device is then demonstrated to restore the motility of the esophagus to effectively transport liquid and solid cargos, potentially useful for patients with esophageal cancer or stricture. Figure 6E shows that the liquid and solid are transported inside an esophagus phantom after being deployed inside a phantom made of silicone (Elastic 50A, Formlabs). The process is visualized with an endoscope when a magnetic field of ≈55 mT and 2 Hz is applied. To deploy our metal stent with integrated magnetic sheets inside a human esophagus, endoscope procedures will be followed when the stent is delivered using the delivery tool used for delivering a conventional metal esophageal stent. The resilience of the magnetic soft sheets allows them to withstand the mechanical stress and still function during the deployment. Lastly, the stent could also be easily monitored by medical imaging such as X-ray imaging in addition to an endoscope. For example, X-ray imaging is used to monitor the magnetic soft sheets which are clearly visible as shown in Figure 6F.

Finally, the stent is demonstrated to allow the transport of large solids to avoid blockage and therein aspiration. In contrast, a traditional esophageal stent has a high risk of food blockage due to the lack of peristalsis. Figure 6G,H prove the effectiveness with control experiments. We show the effectiveness of reducing blockage using the stent integrated with magnetic soft sheets. As shown in Figure 6G, the hydrogel debris in irregular shapes initially block the cross-section of the stent when no magnetic field is applied on the magnetic sheets. The cluster of hydrogel debris starts being squeezed and pumped downward into the stomach phantom when a rotating magnetic field of ≈55 mT at 2 Hz is applied. We further demonstrate the transport of large and intact hydrogel beads (diameter: 12 mm) using the stent with integrated magnetic sheets. As shown in Figure 6H, without the artificial peristalsis, the spheres block the esophagus. In contrast, with the restored peristalsis, magnetic soft sheets integrated into the stent enable the large particles to be pumped into the stomach phantom within 45 s when a magnetic field (55 mT, 2 Hz) is applied.

## 3. Conclusion

In summary, we have reported a soft robotic pump that is fully wireless and can transport liquids and solids efficiently. The fundamental undulating motion is realized by programming a magnetic sheet with a specific magnetization profile. Peristalsis motion is further encoded by designing different magnetization profiles in neighboring sheets. Different designs have been systematically investigated to optimize the pair-wise coordination for both liquid and solid pumping. Finally, we have demonstrated creating an esophageal stent as an example to restore the peristalsis for pumping liquids and solids to prevent blockage and therein aspiration. Our proposed wirelessly actuated robotic pumping mechanism is generic and holds promise in enabling diverse implantable medical devices designed to address the challenges of lumen dysmotility in various diseases such as esophageal cancer. Compared with existing soft robotic pumps, our device is fully wireless which can be seamlessly integrated with existing medical stents. This proposed wireless soft robotic pump marks a significant step toward the development of innovative solutions in the field of soft robotics and point-of-care medical devices.

The proposed undulating motion-based wireless pump may be further improved. First, the magnetic soft sheets could be scaled down using advanced manufacturing methods such as micro-molding^[41]^ to further adapt to narrower lumens. The surface of the magnetic soft sheet could be patterned with microstructures^[42]^ and omni-phobic material coatings^[43,44]^ for improving the transport of various fluids and solids. In addition, the current prototype is actuated with a permanent magnet mounted on a motorized rotational stage. Typically, a person can tolerate magnetic fields up to 7 Tesla,^[45,46]^ whereas our magnetic field measures ≈55 mT which is considerably lower and thus deemed safe. To facilitate efficient pumping, we generally require a magnetic field ranging from 40 to 60 mT. There is potential to further minimize the magnetic field by increasing the magnetic moment of the magnetic module and reducing material elastic modulus.^[47]^ For future applications at home, the esophageal stent could be actuated by a wearable magnetic actuation system which could be developed for long-term actuation of the robotic esophageal stent.

In addition, our proposed wireless undulating motion-based pump mechanism is generic for various medical stents to restore the muscular transport inside the lumens of the human body. For example, the fallopian tubes, also known as the oviduct, are part of the female reproductive system and play a crucial role in transporting eggs from the ovaries to the uterus.^[48,49]^ The peristalsis motion of the fallopian tube muscle may also be impaired in pelvic inflammatory disease^[50]^ due to genetic issues which also cause issues of transporting eggs.^[4,51]^ The same magnetic soft sheets could be integrated into medical stents inside the fallopian tube for transporting eggs from the ovaries to the uterus. In addition, it could also be integrated with airway stents^[52]^ for transporting excessive mucus. Therefore, by emulating and restoring the natural peristalsis motion, our proposed mechanism and device could address the current challenge of lumen dysmotility.

## 4. Experimental Section

### Fabrication of Magnetic Modules and Back Layer:

To fabricate the magnetic modules, NdFeB micromagnets were first mixed thoroughly with Ecoflex 00–30 (Smooth-on Inc.) with a ratio of 1:2 by weight. Then, four layers of Polyester (PET) tapes with a thickness of 65 μm for each layer were used as spacers to construct a thin wall on the edges of an acrylic substrate. The liquid mixture of the Ecoflex 00–30 and NdFeB micromagnets were spread out slowly to reduce the formation of air bubbles. A sharp razor blade was used to scrape the composite materials to ensure the uniform distribution of the mixture on the substrate. Subsequently, the substrate together with the mixture composite was cured at a temperature of 70 °C on a hot plate for 1 h. Lastly, a UV laser cutter (LPKF U4, LPKF AG) was used to cut the cured sheet into a 12 by 6 matrix of magnetic modules with the dimensions of 3 mm by 2 mm by 0.2 mm. Each matrix was cut using a “hinge” method with longitudinal deeper cuts and latitudinal shallower cuts to allow easy magnetization and assembly of the magnetic modules. Additionally, to fabricate the back layer, a separate acrylic glass with four layers of PET tape as boundaries on the edges was used as a substrate to spread out pure Ecoflex 00–30 uniformly and cured at a temperature of 70 °C on a hot plate for 1 h.

### Magnetizing of the Magnetic Modules:

To magnetize the modules, a strip of magnetic modules (12 modules) cut by the laser machine were first wrapped around a magnetizing fixture consisting of a cylinder fixed between two side walls bonded to a glass slide. The fixture was a simple cylinder marked with a π∕4 angular increment and a double-sided adhesive tape layered on the outside of the cylinder to allow the strip to adhere onto it. The fixture was bonded on a glass slide using double-sided adhesive tape to secure the orientation of the fixture. When wrapping the strip around the cylinder, the magnetic modules were placed to not overlap with each other, ensuring proper magnetization of the array. Subsequently, this fixture was placed inside an impulse magnetizer (IM-10-30, ASC Scientific) and a magnetic field impulse of 2.3 T was then applied at a maximum discharging voltage of 300 V. Each subsequent strip was placed on the cylinder with an angular increment on the cylinder to induce a desired incremental phase shift.

### Assembly of the Magnetic Soft Sheets:

The magnetized modules with the desired orientation were bonded to the back layer made of cured Ecoflex 00–30. A 1-mm distance was maintained between neighboring modules while the modules were aligned with the longer edge parallel to each other. Uncured Ecoflex 00–30 was used as an adhesive to bond the magnetic modules to the Ecoflex 00–30 back layer. Once all the magnetic modules were bonded to the Ecoflex 00–30 back layer, the sheet was cut out according to a desired dimension of the joint and the fin. For example, a width of 0.5 mm was selected for both the joint and the fin. In addition, a 4-mm wide extra back layer was kept, extending the joint to further allow secured bonding of the sheet onto the base layer.

### Integration of Magnetic Soft Sheets on a Silicone Stent:

Multiple magnetic sheets were first bonded onto a base layer to form a magnetic sheet blanket by bonding the sheets using uncured Ecoflex 00–30. The blanket was then rolled into a magnetic sheet cylinder with the two edges bonded using uncured Ecoflex 00–30. After curing, the cylindrical structure was tested by being compressed into a flat structure to check for durability. Upon ensuring the bonding strength of all the magnetic sheets on the cylindrical base layer, freshly prepared Ecoflex 00–30 was applied onto the inner surface of the prepared silicone stent. The prepared magnetic sheet cylinder was subsequentially compacted and inserted into the stent. The compression on the magnetic sheet cylinder was removed once the cylinder was inside the stent. All the gaps between the magnetic sheet cylinder and the stent inner surface were ensured to have uncured Ecoflex 00–30 by compression. Lastly, the assembled stent was heated to complete the bonding.

### Integration of Magnetic Sheets on a Meshed Metal Stent:

Similar to “Integration of magnetic soft sheets on a silicone stent,” magnetic soft sheets were bonded to a meshed metal stent (16 × 70 ALIMAXX-ES Fully Covered Esophageal Stent, Merit Medical) while ensuring the back layer integrity. As the metal stent had a varying diameter that was thinner in the middle and wider at the two ends, glycerol was applied for lubrication when placing the magnetic sheet cylinder inside. In the process, 0.1–0.2 mL of glycerol helped lubricate the inner surface and allow for an easier decompression and installation of the magnetic sheet cylinder. Subsequently, a spatula was used to apply sufficient Ecoflex 00–30 first starting from the middle and then inching toward the edges to further seal the edges. After curing the Ecoflex 00–30 at a temperature of 70 °C on a hot plate for 30 min, the durability of the assembled stent was checked by performing the stent compression test.

### Preparation of an Esophagus Phantom:

The esophagus phantom was prepared by 3D printing the segmented parts of a human upper gastrointestinal model. The phantom model was imported and segmented in Autodesk MeshMixer (Autodesk Research) and Fusion360 (Autodesk Research). The segmented parts were printed using a 3D printer (Form 3+, Formlabs Inc.) with UV curable resin Elastic 50A. The 3D printed parts were assembled and bonded by applying UV curable resin Elastic 50A under UV exposure.

### Magnetic Actuation Setup:

The magnetic actuation setup was composed of a step motor (NEMA 17) controlled by a step motor driver (L298N, HiLetgo) using an embedded controller (Arduino Uno). Two neodymium-iron-boron magnets (1 inch by 1 inch by 1 inch, N52) were fixed in a 3D printed fixture using Polylactic acid (PLA) and rotated about the metal shaft connected to the 3D printed fixture for generating a rotating magnetic field with an average amplitude *B* ≈55 mT and frequency *f* from 0.1 to 2 Hz.

### Preparation of Viscous Liquids and Solid Cargos:

The viscous liquids used included syrup, glycerol, and porcine mucus. To make the porcine mucus, mucin (Chem-impex International Inc.) was mixed with water according to a weight ratio of 7 to 1. The viscosities of the liquids were measured by a rheometer (Bonvoisin Digital Rotary Viscometer). The solid cargos used were hydrogel spheres with different diameters (3–5 mm).

## Supplementary Material

Movie S1

Movie S3

Movie S4

Movie S2

Supplementary Information

## Data Availability

The data that support the findings of this study are available in the Supporting Information of this article.
