# Supplementary material for: Wireless Peristaltic Pump for Transporting Viscous Fluids and Solid Cargos in Confined Spaces
Source: Adv Funct Mater. Author manuscript; Available in PMC 2025 Feb 21. (PMC11845220; doi:10.1002/adfm.202405865)
Supplement: Supplementary Information [file NIHMS2011196-supplement-Supplementary_Information.pdf]

ADVANCED FUNCTIONAL MATERIALS

Supporting Information

for *Adv. Funct. Mater.*, DOI 10.1002/adfm.202405865

Wireless Peristaltic Pump for Transporting Viscous Fluids and Solid Cargos in Confined Spaces

*Saksham Sharma, Laura Caroline Jung, Nicholas Lee, Yusheng Wang, Ane Kirk-Jadric, Rishi Naik and Xiaoguang Dong**

Supplementary Information for

Wireless Peristaltic Pump for Transporting Viscous Fluids and Solid Cargos in Confined Spaces

Saksham Sharma[1], Laura Caroline Jung[1], Nicholas Lee[1], Yusheng Wang[1,4], Ane Kirk-Jadric[1], Rishi Naik[5], Xiaoguang Dong[1,2,3,4]*

[1] Department of Mechanical Engineering, Vanderbilt University, TN, 37212, US
[2] Department of Biomedical Engineering, Vanderbilt University, TN, 37212, US
[3] Department of Electrical and Computer Engineering, Vanderbilt University, TN, 37212, US
[4] Vanderbilt Institute for Surgery and Engineering, Vanderbilt University, TN, 37212 US
[5] Vanderbilt School of Medicine, Vanderbilt University, TN, 37240, US

* Corresponding to xiaoguang.dong@vanderbilt.edu

The PDF file includes:

Figs. S1- S6

Table S1

Notes for Movies S1 to S4

Other Supplementary Material for this manuscript includes the following:

movies S1 to S4

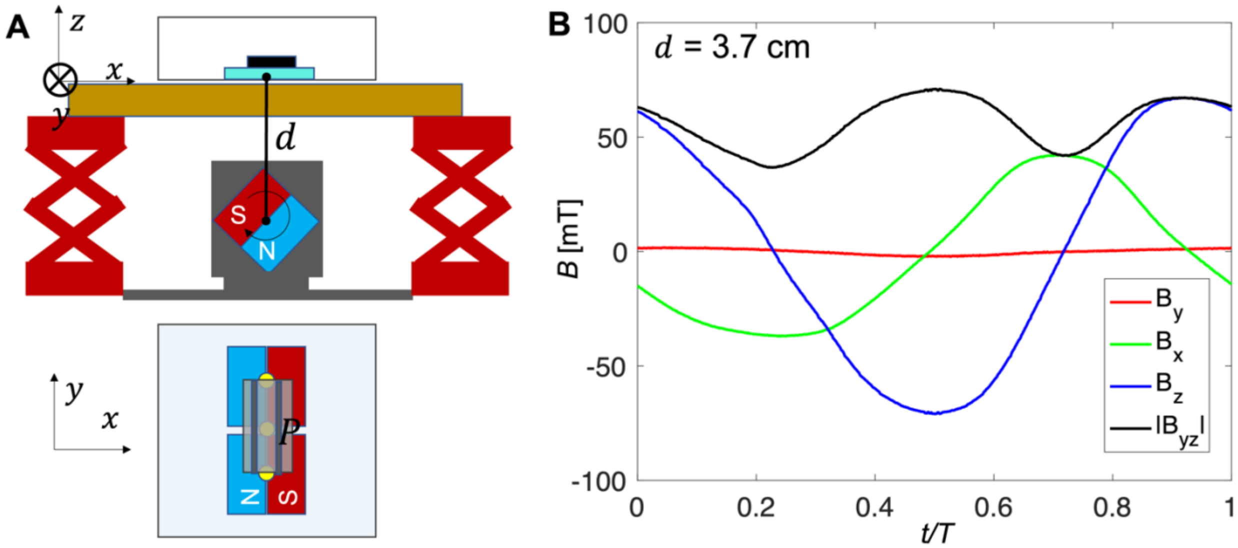

Fig. S1 Characterization of the magnetic field waveform. A. Illustration of the measurement of the magnetic field. B. Example measurement of the magnetic field at a given location ($d = 3.7$ cm).

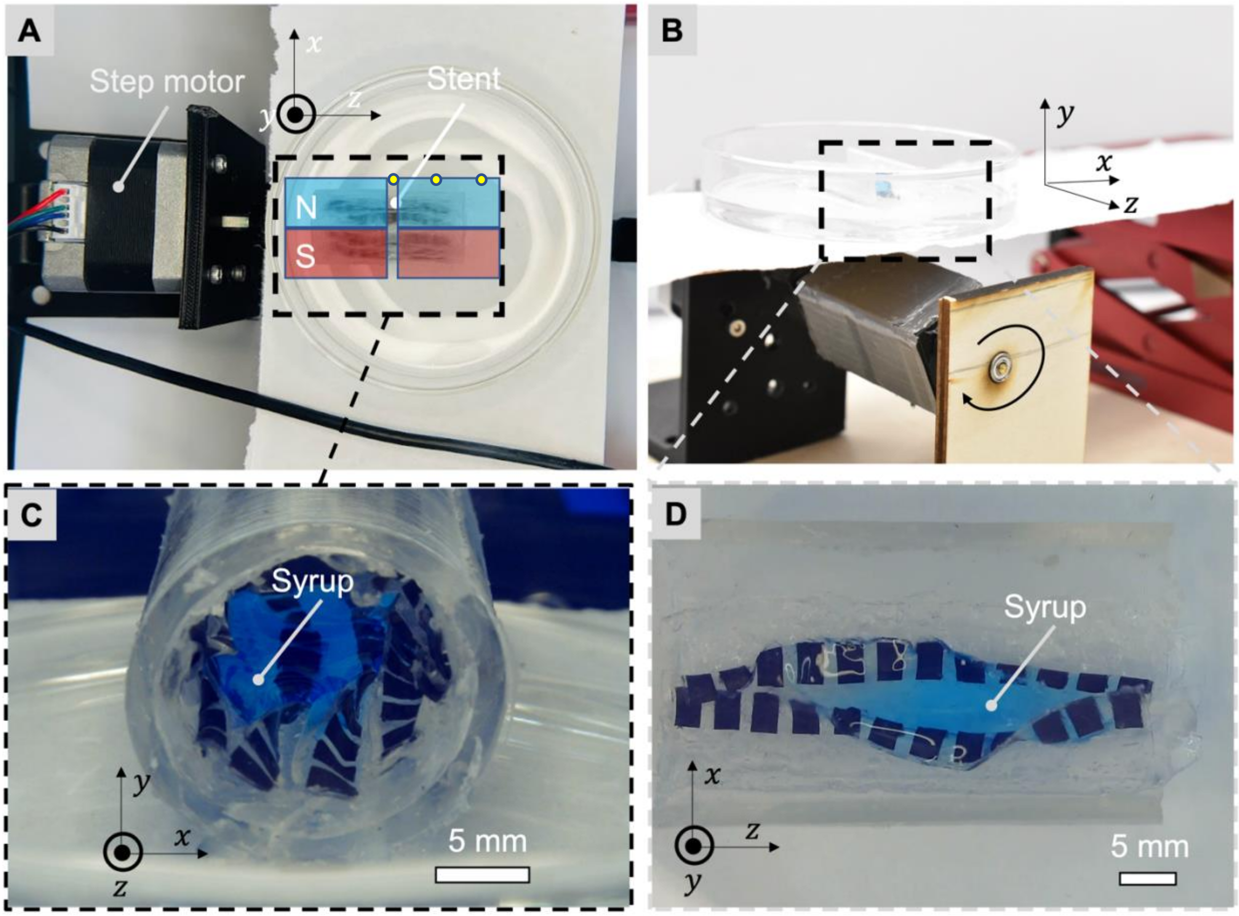

Fig. S2 Experimental setup for magnetically actuating the stent. A. Optical image of the experimental setup in a top view. B. Optical image of the experimental setup in a perspective view when testing the pair-wise coordination of two magnetic sheets. C. Zoomed in optical image of a silicone stent with magnetic soft sheets integrated for pumping syrup. D. Optical image of a pair of magnetic soft sheets pumping syrup.

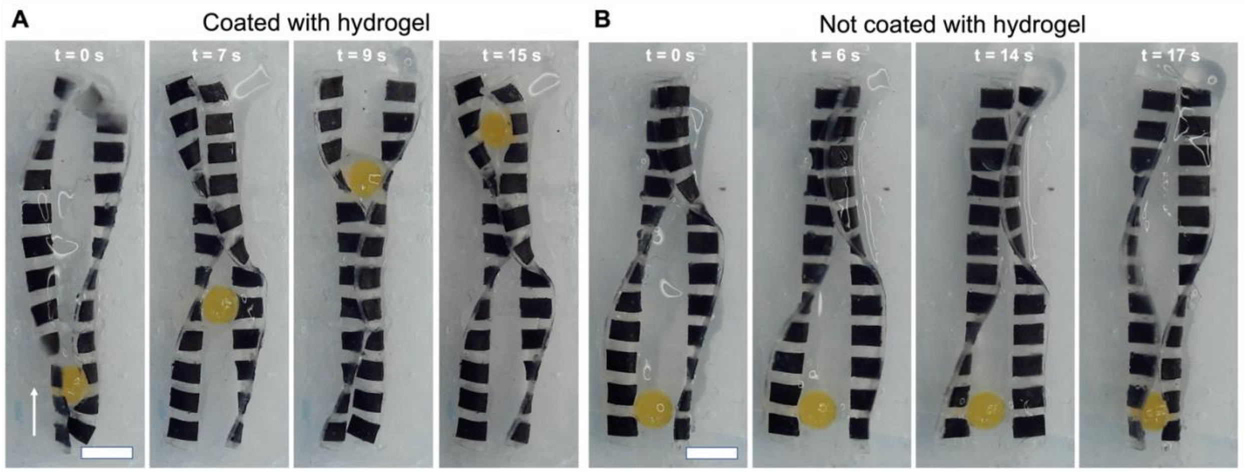

Fig. S3 Pumping speed difference for a pair of sheets with or without hydrogel coating. A. Pair-wise transportation of hydrogel sphere with coated magnetic sheets. B. Pair-wise transportation of hydrogel sphere with non-coated magnetic sheets. Scale bars, 5 mm.

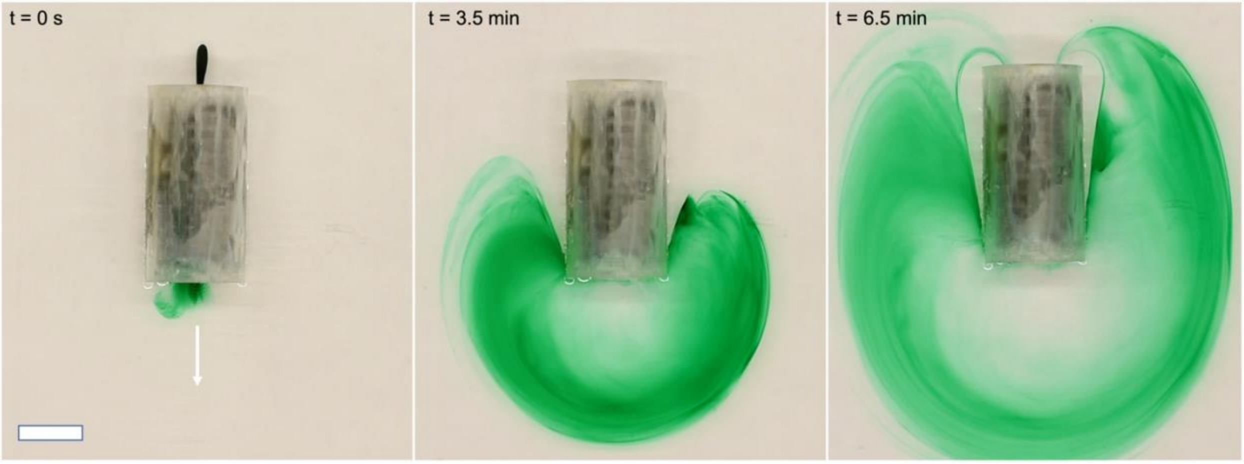

Fig. S4 Flow wake for a silicone stent with magnetic sheets pumping glycerol while visualized by green dye. The stent is placed in a horizontal plane inside a container filled with viscous fluid (pure glycerol, dynamic viscosity: 983 mPa·s). White arrow indicates the pumping direction. Scale bar, 10 mm.

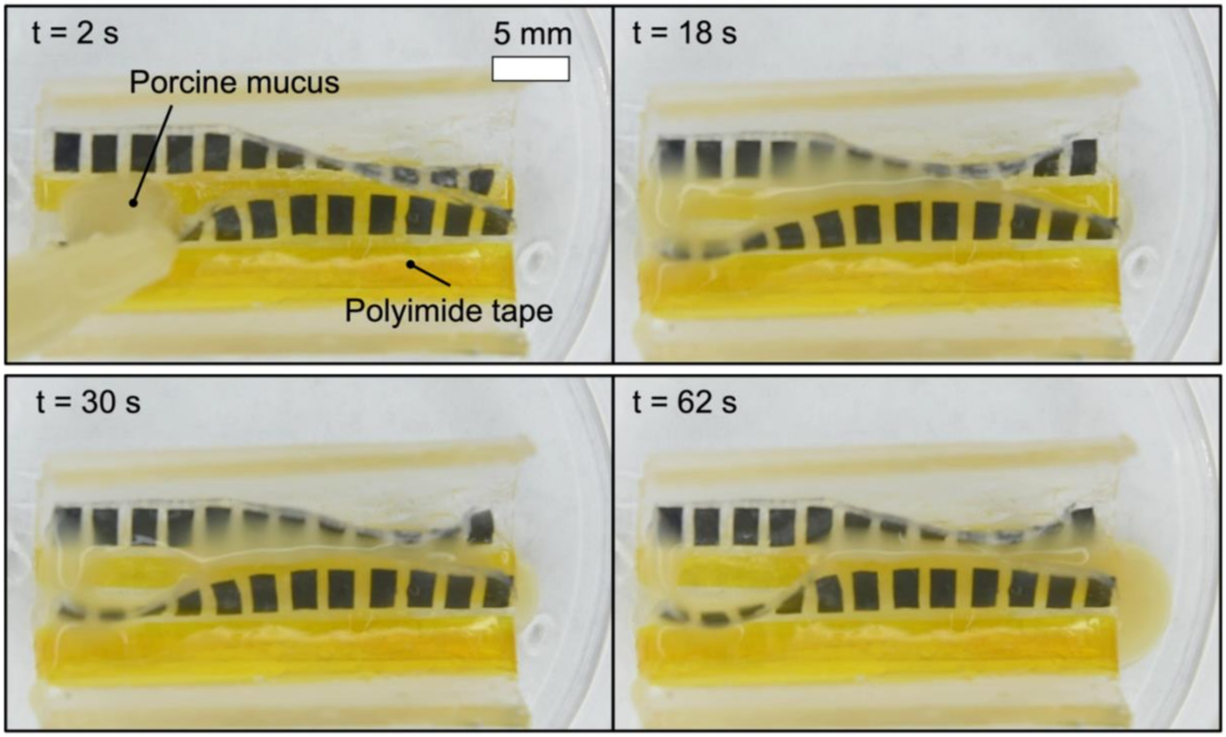

Fig. S5 Transportation of porcine mucus by a pair of magnetic soft sheets. Phase shift $\Delta\phi = -3\pi/4$.

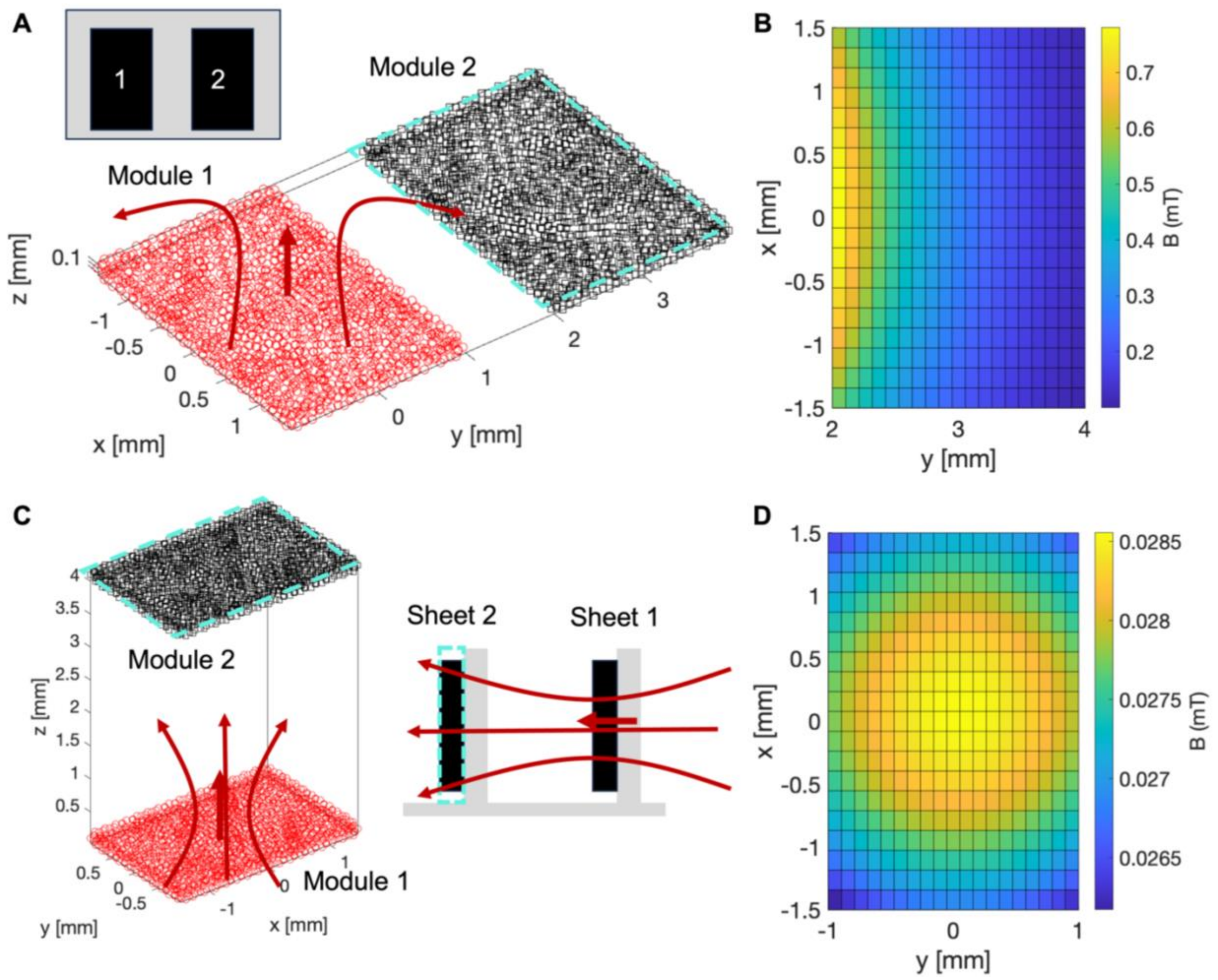

Fig. S6. Estimation of the magnetic field generated by neighboring modules and sheets. A. Illustration of the magnetic field generated at the location of a magnetic module by a neighboring magnetic module. B. Magnetic field distribution using a distributed magnetic dipole model for the configuration in A. C. Illustration of the magnetic field generated at the location of a magnetic module by the closest magnetic module in a neighboring sheet. D. Magnetic field distribution using a distributed magnetic dipole model for the configuration in C.

Table S1. Parameters of the silicone stent with magnetic sheets.

Parameter	Name	Value
$\boldsymbol{L_m}$	Magnetic module length	3 mm
$\boldsymbol{w_m}$	Magnetic module width	2 mm
$\boldsymbol{t_m}$	Magnetic module thickness	0.2 mm
$\boldsymbol{\phi}$	Magnetic moment	$\frac{j\pi}{6}, j = 1,2,\ldots,N$
$\boldsymbol{M}$	Magnetization	61.9 kA/m
$\boldsymbol{E_m}$	Youngs modulus of the magnetic module	144 kPa
$\boldsymbol{E_b}$	Youngs modulus of the back layer	30 kPa
$\boldsymbol{t_{joint}}$	Joint thickness	0.2 mm
$\boldsymbol{t_{fin}}$	Fin thickness	0.2 mm
$\boldsymbol{L_{joint}}$	Joint length	4 mm
$\boldsymbol{L_{fin}}$	Fin length	0.5 mm
$\boldsymbol{d_m}$	Spacing between magnetic modules	1 mm
$\boldsymbol{N}$	Number of magnetic modules in a sheet	12
$\boldsymbol{n}$	Total number of sheets in a stent	10
$\boldsymbol{D_i}$	Inner diameter of a stent	15.5 mm
$\boldsymbol{D_o}$	Outer diameter of a stent	16.5 mm

Supplementary Videos.

Movie S1 Single sheet undulating motion.

This video shows the undulating motion of a single magnetic sheet.

Movie S2 Pair-wise undulating motion and transportation of particles.

This video shows the transportation of particles and liquids.

Movie S3 Transportation of liquid and solid in a silicone stent.

This video shows the transportation of particles and liquids in a silicone stent with integrated magnetic sheet.

Movie S4 Esophageal stent for pumping liquids and solids.

This video shows the transportation of particles and liquids by the esophageal stents.
